# Numerous Serine/Threonine Kinases Affect Blood Cell Homeostasis in *Drosophila melanogaster*

**DOI:** 10.3390/cells13070576

**Published:** 2024-03-26

**Authors:** Sebastian Deichsel, Bernd M. Gahr, Helena Mastel, Anette Preiss, Anja C. Nagel

**Affiliations:** 1 Department of Molecular Genetics, Institute of Biology, University of Hohenheim, 70599 Stuttgart, Germany; 2 Institute of Biology, University of Hohenheim, 70599 Stuttgart, Germany

**Keywords:** blood cell homeostasis, crystal cell, *Drosophila melanogaster*, hematopoiesis, protein phosphorylation, phospho-status, Ser/Thr kinase, signaling network

## Abstract

Blood cells in *Drosophila* serve primarily innate immune responses. Various stressors influence blood cell homeostasis regarding both numbers and the proportion of blood cell types. The principle molecular mechanisms governing hematopoiesis are conserved amongst species and involve major signaling pathways like Notch, Toll, JNK, JAK/Stat or RTK. Albeit signaling pathways generally rely on the activity of protein kinases, their specific contribution to hematopoiesis remains understudied. Here, we assess the role of Serine/Threonine kinases with the potential to phosphorylate the transcription factor Su(H) in crystal cell homeostasis. Su(H) is central to Notch signal transduction, and its inhibition by phosphorylation impedes crystal cell formation. Overall, nearly twenty percent of all *Drosophila* Serine/Threonine kinases were studied in two assays, global and hemocyte-specific overexpression and downregulation, respectively. Unexpectedly, the majority of kinases influenced crystal cell numbers, albeit only a few were related to hematopoiesis so far. Four kinases appeared essential for crystal cell formation, whereas most kinases restrained crystal cell development. This group comprises all kinase classes, indicative of the complex regulatory network underlying blood cell homeostasis. The rather indiscriminative response we observed opens the possibility that blood cells measure their overall phospho-status as a proxy for stress-signals, and activate an adaptive immune response accordingly.

## 1. Introduction

The hematopoietic system in *Drosophila melanogaster* serves primarily immune responses, as oxygen disperses from the trachea via the hemolymph to the various organs. There are three distinct blood cell types or hemocytes fulfilling the immunity function, plasmatocytes, crystal cells and lamellocytes. Plasmatocytes constitute the majority of blood cells with more than 90% in a healthy animal. They are bone fide phagocytes resembling mammalian macrophages, able to engulf and destroy pathogens and cellular debris, cells infected with viruses and apoptotic cells. Moreover, they secrete antimicrobial peptides and produce extracellular matrix during wound healing (for review [1,2,3]). In addition, plasmatocytes are central to blood cell homeostasis. They have the capability of self-renewal, i.e., regenerating and increasing the blood cell pool, thus serving as the source for pro-hemocytes. Moreover, the other two cell types may arise by trans-differentiation directly from plasmatocytes (for review [4]).

Crystal cells make up 2–5% of the total hemocytes, whereas lamellocytes differentiate particularly in response to endo-parasitic wasp infestation of larvae. Crystal cells share similarities with mammalian megakaryocytes. They also combat microorganisms, are important for wound healing and serve the hypoxic response. They are named for the para-crystalline inclusions of pro-phenoloxidases (PPOs), which are released into the hemolymph in stress situations, where they induce a melanization reaction and the formation of reactive oxygen species that eventually kill invaders (for review [1,2,3]).

*Drosophila* hematopoiesis starts in a first wave with embryonic hemocyte precursors that originate in the head mesoderm. They disperse throughout the embryo, cease proliferation and differentiate to plasmatocytes and crystal cells. In the larva, hemocytes colonize their niches in the peripheral larval body wall, the so-called hematopoietic pockets, representing the sessile compartment. There, plasmatocytes self-renew in response to local, neuronal and systemic signals [5,6,7]. Moreover, plasmatocytes may trans-differentiate to crystal cells, and in case of parasitism to lamellocytes, hence providing the source of all blood cell types [8,9,10,11,12]. In addition, hemocytes circulate in the hemolymph, which is in a dynamic steady state with the sessile compartment, as there is a constant exchange (for review [4]). The second hematopoietic wave occurs in the lymph gland, a true hematopoietic organ that originates in the embryonic cardiogenic mesoderm, and fully develops only during larval stages. Lymph gland plasmatocytes and crystal cells are released during pupariation to serve the imago’s innate immune responses. Parasitization of the larva, however, induces massive proliferation and differentiation of lamellocytes, and a premature burst of the lymph gland, releasing all blood cells into the hemolymph to fight off the invader (for review [1,2,3,4]).

**Figure 1:**
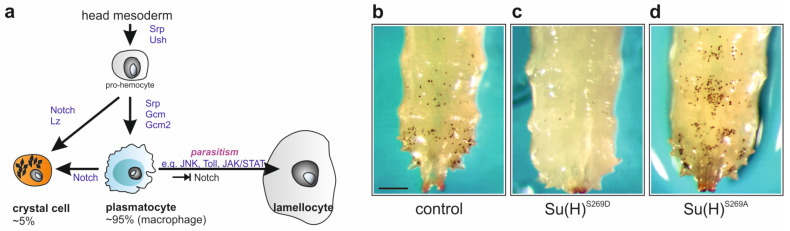
Blood cell homeostasis in *Drosophila*. (**a**) Scheme of larval blood cell development. Precursors, called pro-hemocytes, are determined in the embryonic head mesoderm by the activity of Serpent (Srp) and U-shaped (Ush). Pro-hemocytes are able to proliferate, and to differentiate into predominant plasmatocytes under the influence of Glial cells missing transcription factors (Gcm/Glide, Gcm2). The alternative crystal cell fate is induced by Notch activity via Lozenge (Lz); it can also arise by trans-differentiation of plasmatocytes [3,4]. Lamellocytes differentiate in response to parasitism, involving the activity of several signaling pathways, including JNK, Toll, JAK/STAT and the repression of Notch activity [3]. (**b**–**d**) Sessile crystal cells can be detected through the cuticle of heated larvae [48,49]. Control larvae (*Su(H)^gwt^*) display around 100 crystal cells in the dorsal hematopoietic pockets of the last two posterior segments (**b**). The phospho-mimetic allele *Su(H)^S269D^* barely develops any crystal cells (**c**), whereas the phospho-mutant *Su(H)^S269A^* displays increased numbers (**d**) [36]. Representative images are shown. Scale bar, 250 µm.

Many molecular details of *Drosophila* hematopoiesis have been uncovered in the past (Figure 1a). Briefly, pro-hemocyte fate is specified by the GATA-type transcription factor Serpent (Srp) and the Friend of GATA homolog U-shaped (Ush). Plasmatocytes are then determined by the transcription factors Glial cells missing (Gcm/Glide and Gcm2) [13,14]. Interestingly, the major signaling pathways controlling hematopoiesis are downstream targets of Gcm transcription factors, including Notch, Hedgehog, Wnt, FGFR and JAK/STAT [15]. Proliferation and maturation of plasmatocytes, however, is under the influence of a multitude of external and internal cues, including for example nutritional status, a tumorous environment, injury or reactive oxygen species [3,16,17,18,19,20,21,22,23,24,25,26,27].

Crystal cell fate relies on the activity of the AML-1/RUNX homologue Lozenge (Lz) that functions together with Srp, but is restricted by Ush. Hence, the three together control the number of crystal cells as well as their maturation (for review [3,28,29,30,31,32]). As Lz is regulated by the Notch signaling pathway, crystal cell fate, maturation and survival strictly depend on Notch activity [33,34,35]. Moreover, trans-differentiation of plasmatocytes to crystal cells relies on Notch signaling activity as well [9]. Finally, the differentiation of lamellocytes from plasmatocytes, initiated by parasitoid wasp infestation, is induced by the combined activity of several pathways including JNK, Toll, EGFR, JAK/STAT and Ecdysone pathways as well as the inhibition of the Notch pathway (for review [3]).

In our previous work, we have uncovered a novel regulatory mechanism of Notch signaling activity in the context of blood cell homeostasis, involving the CSL gene regulator Suppressor of Hairless (Su(H)) [36]. CSL is the acronym for human CBF1 (*C-promoter Binding Factor 1*, corresponding to mammalian RBPJ, Recombination signal Binding Protein), *Drosophila melanogaster* Su(H) and *Caenorhabditis elegans* Lag1 (Lin-12 and glp-1). CSL is the singular, central transcription factor transmitting Notch signals, and is conserved from invertebrates to vertebrates [37,38,39,40,41,42,43,44]. In *Drosophila*, Su(H) protein can be phosphorylated at Serine 269, which impedes its DNA-binding capability [45]. This phosphorylation was observed in cells of hemocyte origin, and may alter blood cell homeostasis affecting crystal cell numbers [36,45,46]. In fact, in the knock-in allele *Su(H)^S269D^* mimicking permanent phosphorylation, crystal cell formation was blocked to near completion, whereas the corresponding phospho-deficient *Su(H)^S269A^* allele displayed increased crystal cell numbers [36] (Figure 1b–d). Aiming at the identification of the kinases involved, we analyzed Ser/Thr kinases with the potential to target Su(H) Ser269. We identified Pkc53E involved in Su(H) phosphorylation in response to larval parasitization, however, regulating blood cell homeostasis in normal conditions as well [47]. In the course of the work presented here, about 20% of the known *Drosophila* Ser/Thr kinases were examined for their role in blood cell formation. Two assays were employed, global and blood cell-specific overexpression and downregulation, respectively. Unexpectedly, the vast majority of the investigated Ser/Thr kinases of any kinase class influenced crystal cell homeostasis. The contribution of protein kinases to hematopoiesis has remained fairly understudied to date, despite their defined roles in the various hematopoietic signaling pathways. However, not only kinases that are central core components of respective signaling cascades influenced blood cell formation. Instead, the rather general involvement of many kinases points to a much more complex regulation of blood cell homeostasis than anticipated so far. Perhaps, blood cells measure their overall phospho-status as a proxy for stress-signals, and activate an adaptive immune response accordingly.

## 2. Materials and Methods

### 2.1. Fly Work

Fly strains used in this work are listed in the Supplemental Table S1. Flies were raised on standard fly food at ambient temperature (25 °C). Comparative crosses were set up in parallel. For the ectopic expression of the kinase constructs, the Gal4/UAS system was applied [50]. We either used the ubiquitous driver line da-Gal4 (BL55849) [51] or the hemocyte-specific driver line HmlΔ-Gal4 (BL30141, FBrf0210198; hml-Gal4), which induces transgene expression in hemocytes prior to or at the stage of crystal-cell commitment [34].

Most UAS-kinase strains were obtained from Bloomington Drosophila Stock Center (BDSC) or *FlyORF* (FO) [52] (see Supplemental Table S1). UAS-lines for the overexpression of Alc [53], HipK [54], Par-1 [55], PDK1 [56] and Sik2 [57], respectively, were kindly provided by P. Callaerts (VIB, KU Leuven), U. Walldorf (Saarland University, Homburg), A. Ephrussi (EMBL, Heidelberg), H. Stocker (ETH Zürich) and N. Tapon (Francis Crick Institute, London). Five kinase overexpression strains were generated previously [47] and during this work (see below). To avoid position effects from the chromosomal insertion site, all pUASt-attB constructs were integrated into the identical position at 96E by site-specific recombination as outlined earlier [58]. To this end, DNA constructs were injected into *vasa-ϕC31*; *96EattP* embryos [58]. Transgenic flies were recognized by their red eye color. True-breeding lines were established; they were verified by monitoring PCR and sequencing. Kinase mutants were derived from BDSC (see Supplemental Table S1). The *Drak^del^* deletion was kindly provided by D. Hipfner (IRCM, Montreal) [59]. Lines used for RNAi-mediated downregulation were obtained from either the BDSC or the Vienna Drosophila Resource Center (VDRC) (see Supplemental Table S1).

### 2.2. Cloning of UAS-Kinase Constructs

The cDNAs encoding the respective kinases were obtained from the Drosophila Genome Resource Center (DGRC), cdk7 (FMO01629), Dyrk3 (RE60792), Fray (RE53265) and Gskt (FMO04376). The same scheme was used for tagging and cloning either cdk7 or Gskt. Firstly, the kinase cDNA was amplified from its original vector using oligonucleotides with *Xho* I (5′), and *Xba* I (3′) overlaps. Secondly, it was cloned into a likewise opened pBT vector (Stratagene) harboring 3xHA tags, inserted as annealed respective oligonucleotides into the *Acc* 65I/*Xho* I sites, named pBT-HA-STaRT. The HA-tagged cDNA was then released by *Acc* 65I/*Xba* I digest, to be reinserted into likewise opened pUASt-attB vector [58]. Dyrk3 cDNA was amplified with primers containing *Sal* I/*Bam* HI overhangs, and inserted into likewise opened pBT-HA-STaRT. The HA-tagged Dyrk3 cDNA was then released with *Acc* 65I and *Xba* I and cloned into pUASt-attB [58] as above. In case of Fray, the primers contained *Sal* I/*Eco* RI overhangs. The amplicon was inserted into *Xho* I/*Eco* RI opened pBT-HA-STaRT, released with *Acc* 65I and *Eco* RI and shuttled into pEGFP-N1 (Clontech). After a second release with *Bam* HI/*Xho* I, the insert was shuttled into the *Bgl* II/*Xho* I sites of pUASt-attB [58]. Constructs were verified by diagnostic digests and by sequencing before generating transgenic lines. Primers used for cloning are listed in the Supplemental Table S2.

### 2.3. Quantification of Crystal Cells

Crystal cells were visualized through the larval cuticle as described before [48,49]. Larvae from the respective crosses were reared on normal fly food. Overcrowding was strictly avoided to synchronize the larval stages that were heated in batches as described previously [47]. Dorsal views from the larval posterior end were taken with a Pixera Pro 120ES camera (Pixera, Santa Clara, CA, USA), mounted onto a Wild M3Z stereo microscope (Leica, Wetzlar, Germany) using Pixera Viewfinder 2.5 software. Melanized crystal cells were counted in the last to segments using the ‘Cell Counter’ Plugin of Image J 1.51 (Fiji) software [60]. At least two biological replicates were performed; the total number of analyzed larvae of each genotype is given within the figure.

### 2.4. Selection of Ser/Thr Kinases

Ser/Thr kinases were selected by their potential to recognize the Su(H) S269 target sequence FNRLR**S**QTVSTR by the following two approaches. First, an in silico search with the GPS3.1 software tool yielded 20 *Drosophila* kinase candidates [47,61]. Second, of 245 human kinases, 62 had the capability to in vitro phosphorylate the Su(H) beta-trefoil domain overlapping the S269 target site (ProQinase, Freiburg, Germany) [47]. These human kinases correspond to 40 *Drosophila* kinase candidates, containing ten of the first group. Altogether, 46 kinase candidates were tested in gain and loss of function screens.

### 2.5. Statistical Analyses

For statistic evaluation of the data, we used Microsoft Excel to calculate mean, standard deviation (SD) and median; statistical significance was evaluated by using a two-tailed analysis of variance (ANOVA) approach for multiple comparisons according to Dunnett’s test relative to the control; values were *** *p* ≤ 0.001, ** *p* ≤ 0.01, * *p* ≤ 0.05, and *p* > 0.05 not significant (n.s.). Statistical graphs were created with Excel 2016 (MS Office) and Origin R© 2018b software (OriginLab Corporation, Northampton, MA, USA).

## 3. Results

### 3.1. Experimental Design

**Figure 2:**
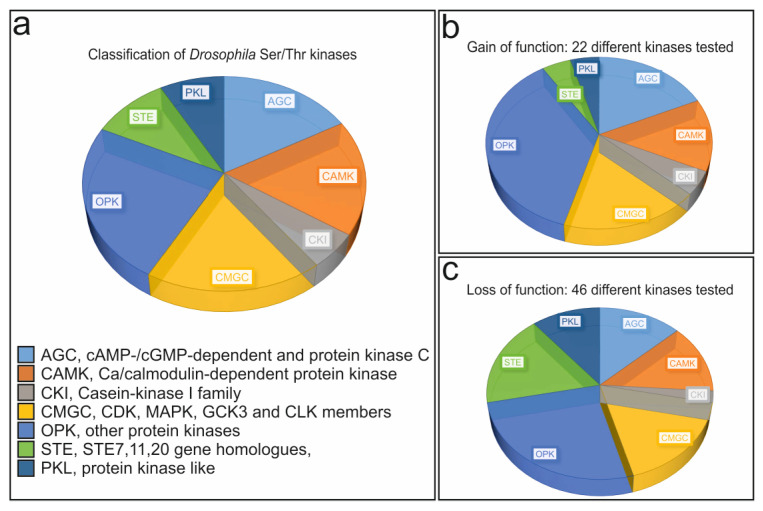
Classification of tested kinases. (**a**) A total of 184 *Drosophila* Ser/Thr kinases are grouped in the seven indicated classes. (**b**) Classification of the 22 kinases tested by overexpression. Note a slight overrepresentation of other protein kinases (OPK). (**c**) Classification of the 46 kinases analyzed in loss of function assays. Note the overrepresentation of STE kinases. Abbreviations: AGC, cAMP-dependent, cGMP-dependent and protein kinase C; CAMK, Ca/calmodulin-dependent protein kinase; CKI, Casein Kinase 1 family; CMGC, CDK, MAPK, GCK3 and CLK members; OPK, other protein kinases; STE, STE7,11,20 gene homologues; PKL, protein kinase like.

Serine/Threonine (Ser/Thr) kinases constitute the largest group of protein kinases. They have been further classified according to their structure, function and targets (Figure 2a) [62,63,64,65,66,67]. In this work, Ser/Thr kinases were selected in silico and in vitro by their potential ability to target the sequence FNRLR**S**QTVSTR corresponding to the Su(H) S269 target sequence [47,61]. This way, 46 kinases were selected, representing roughly 20% of all *Drosophila* Ser/Thr kinases (Table 1).

Two assays were employed, global and hemocyte-specific (1) overexpression and (2) downregulation of a particular kinase, respectively, thereby considering adequate overall class representation (Figure 2b,c). The assays were based on the observation that the phospho-mimetic *Su(H)^S269D^* allele largely fails to produce crystal cells, in contrast to the phospho-deficient *Su(H)^S269A^* allele developing more crystal cells than controls (Figure 1b,c) [36]. Hence, we expected a substantial impact on crystal cell formation upon the activation or the inhibition of a given kinase involved in hematopoiesis. Sessile crystal cells can be visualized in a coarse experiment: crystal cells rupture upon heating of third instar larvae, resulting in a melanization reaction [3,48,49]. The blackened cells are visible through the larval cuticle; they were recorded as an approximation for crystal cell numbers, allowing the quantitative comparison of multiple genotypes (Figure 1b–d).

### 3.2. Influence of Ubiquitous and Hemocyte-Specific Induction of Ser/Thr Protein Kinases on Blood Cell Homeostasis

In the first line of experiments, the effects of an overexpression of Ser/Thr kinases on crystal cell numbers were investigated. In order to determine the specific impact on blood cells and distinguish it from overall effects, we wanted to compare the outcome of a general overexpression and a cell type-specific overexpression. Using the Gal4/UAS system, the respective UAS lines are expressed in a temporally and/or spatially restricted manner [50]. The da-Gal4 line is a ubiquitously expressing driver line, which allows inducing a general expression throughout embryogenesis and early larval life [51]. The HmlΔ-Gal4 (hml-Gal4) driver line, however, drives UAS constructs specifically within differentiated hemocytes prior to or at the stage of crystal cell commitment [34]. Whereas da-Gal4 is expected to induce kinase expression in all tissues, including blood cell precursors, hml-Gal4 is highly specific to determined blood cells. Hence, kinase expression may only alter the path of differentiation but not induce de novo differentiation. A total of 22 different kinases were assayed in the overexpression experiments. Three EP lines and fifteen UAS lines were used in this study (see Supplemental Table S1). In addition, four UAS-overexpression lines were established in the course of this work (Cdk7, Dyrk3, Gskt, and Fray). To this end, the respective cDNAs were PCR-amplified and cloned into a suitable UAS-attB transformation vector [58]. Transgenic lines were established by site-specific recombination, integrating the constructs at the identical chromosomal position to avoid any position effects [58].

**Figure 3:**
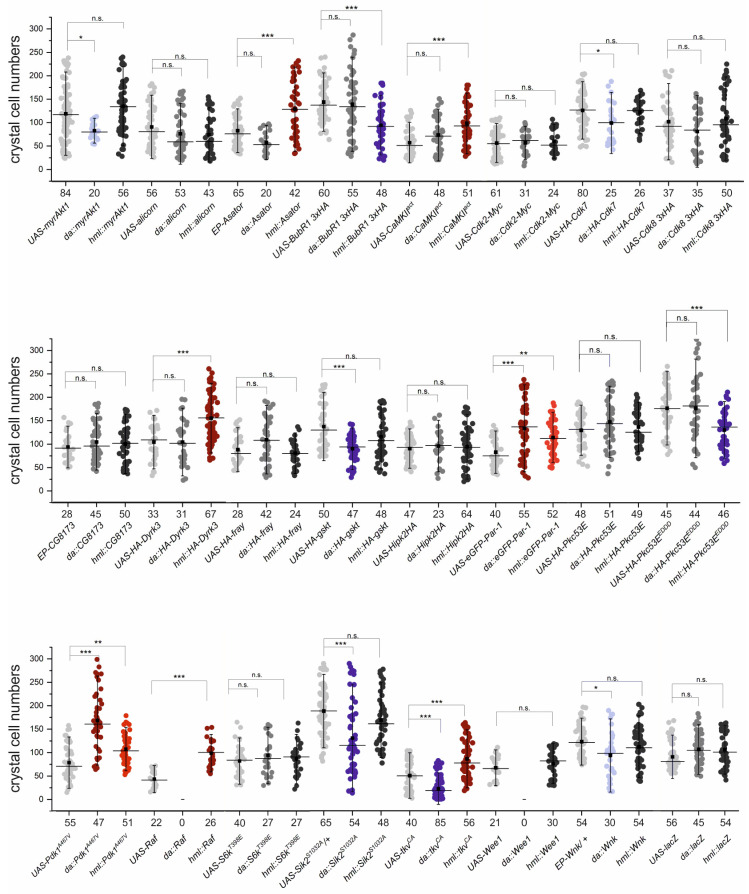
Crystal cell formation in response to ectopic expression of Ser/Thr kinases. Expression of the indicated kinases was induced ubiquitously with da-Gal4, or specifically within hemocytes using hml-Gal4. UAS kinase strains are in alphabetical order (see Table S1). Crystal cells in the dorsal hematopoietic pockets were recorded in the last two segments; every dot represents one larva (n, as shown below the X-axis). UAS-lacZ served as a control. Crystal cell increase is indicated in red and decrease in blue; coloration reflects significance. Whiskers show standard deviation, center line the median and center dot the average. ANOVA for multiple comparisons according to Dunnett’s test relative to the respective UAS line was employed (*** *p* ≤ 0.001, ** *p* ≤ 0.01, * *p* ≤ 0.05, n.s. *p* > 0.05 not significant).

For the assay, control and experimental crosses were set up in parallel; the respective UAS-kinase line itself served as control to be compared with the effects of a global or a hemocyte-specific ectopic expression. We hypothesized that the effects of global overexpression may reflect a more general activity, e.g., on proliferation, whereas hemocyte-specific effects may reflect a more specific role during hematopoiesis and during hemocyte differentiation, respectively. The results are shown in Figure 3, allowing to classify the kinases into inhibitors or mediators of crystal cell formation, and/or kinases with a more general role.

Only two kinases impeded crystal cell formation significantly when specifically expressed in hemocytes, BubR1 and the activated isoform of Pkc53E^EDDD^. We recently showed that Pkc53E is indeed involved in the phosphorylation of Su(H) at S269, in accordance with the observed inhibition of crystal cell formation [47]. BubR1, however, has several essential functions during mitosis, and the overexpression may affect mitotic timing of hemocytes [68]. A loss of crystal cells was also seen upon the ubiquitous but not the hemocyte-specific induction of Gskt and Sik2^S1032A^. The former shares similarities with GSK3 kinase, a core component of the Wnt signaling pathway, whereas the latter regulates energy homeostasis as well as Notch signal transduction [57,69,70]. Hence, both may have a more general role in proliferation and development. The mild effects of a global overexpression of Akt1, Cdk7 and Wnk may be explained likewise [71,72,73,74,75,76,77]. In contrast, the activated Pdk1^A467V^ and Par-1 kinases caused additional crystal cells in both the global and the hemocyte-specific overexpression situations, whereas three kinases, Asator, CaMKII^act^ and Dyrk3, induced a hemocyte-specific increase in crystal cell numbers only (Figure 3). Pdk1 is a master kinase with a crucial role in cell growth [56,78,79], easily explaining the general increase in cell numbers. Par-1, however, plays an important role in cell polarization and tumor suppression, and balances proliferation by influencing the Hippo signaling pathway [80,81,82]. Asator is involved in regulating microtubule spindle function, and may hence relate to cell cycle control [66,83], whereas both Dyrk3 and CaMKII have been connected to calcium signaling as well as to *Drosophila* hematopoiesis [22,84,85]. A special case is represented by the activated Tkv^CA^ construct; the global overexpression induced a reduction in crystal cells, whereas the hemocyte-specific expression resulted in increased numbers (Figure 3). Tkv encodes a Dpp receptor subunit, i.e., acts as a core component within the TGF-beta signaling cascade involved in pattern formation and development [86,87,88]. Perhaps the global overexpression somehow interferes with the development of the embryonic head mesoderm or with cell migration, whereas the hemocyte-specific activation in the larvae acts agonistically [89,90,91,92]. The global overexpression of Raf and Wee1, however, was lethal, whereas hemocyte-specific overexpression of the former increased crystal cell numbers. This result is expected since Raf is the central core component of RTK signaling pathways and hence expected to profoundly affect cell growth and survival, and induce proliferation when overactive [74,93,94,95,96]. The Wee kinase, in contrast, blocks entry into mitosis. Accordingly, a general overexpression may block development altogether, whereas induction in specified hemocytes appears without consequences [74,97,98,99]. In contrast, neither global nor hemocyte-specific overexpression of the UAS-lacZ control had an impact on crystal cell numbers (Figure 3). Together, these results show that a majority of kinases somehow influenced crystal cell formation, albeit only a few had been directly associated with hematopoiesis in the past. Presumably, these effects are largely indirect and not a result of a Su(H) S269 phosphorylation, rather reflecting the intricate cross-talk amongst signaling pathways via kinase activity.

### 3.3. Downregulation of Kinase Activity Reveals a General Involvement of Ser/Thr Kinases in Crystal Cell Formation

Surprised by the broad impact of the overexpression of Ser/Thr kinases on crystal cell development, we sought to analyze the effects of a knockdown of kinase activity. The rationale was that an overexpression of Ser/Thr kinases might be not very specific since kinases are known to cross-react and phosphorylate targets with a decent similarity [64,65,66,67,100]. Moreover, not all of the overexpressed kinases were present in an activated form; hence, we may have missed some in the previous assay. Accordingly, we doubled the number of kinases to be analyzed in a loss of function screen to a total of 46 (see Table 1).

**Figure 4:**
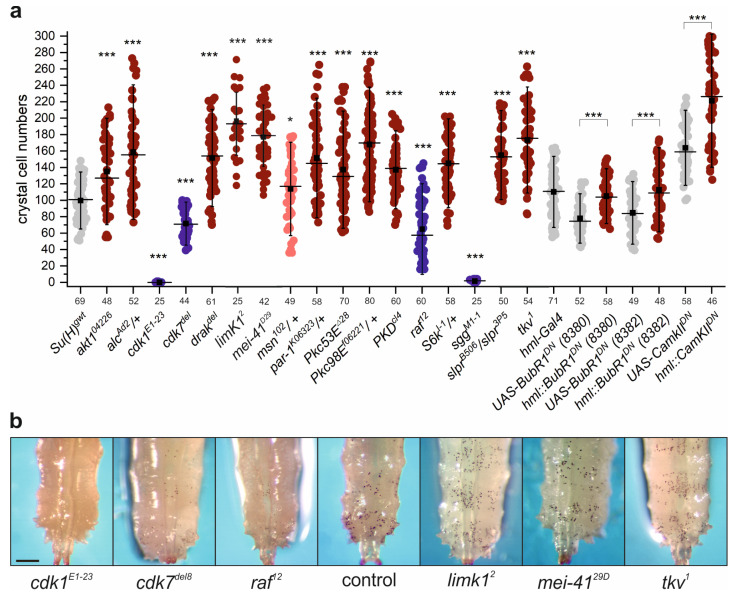
Crystal cell numbers are altered in kinase mutants. (**a**) Crystal cell numbers registered in larvae mutant for the indicated kinase or with kinase activity blocked in hemocytes. Numbers represent crystal cells in dorsal hematopoietic pockets from the last two segments. Every dot represents one larva (n, as shown below X-axis). Crystal cell increase is indicated in red and decrease in blue; intensity reflects significance. . Whiskers, standard deviation; center line, median; center dot, average. For statistical analysis, ANOVA for multiple comparisons according to Dunnett’s test relative to control was employed (*** *p* ≤ 0.001, * *p* ≤ 0.05). *Su(H)^gwt^* served as a control in case of the mutants, and the respective UAS strain in case of the dominant negative lines. (**b**) Representative examples for mutant larvae of the indicated genotype. Left to the *Su(H)^gwt^* control are mutants with reduced, and to the right mutants with increased crystal cell numbers in alphabetical order.

We were able to screen 17 kinase mutants for crystal cell formation (Figure 4). However, not all developed into third instar larvae, and five could only be investigated as heterozygotes (*alc^Ad2^*, *msn^102^*, *par-1^k06334^*, *Pkc98E^f06221^, S6K^I-1^*). The kinase mutants displayed significant changes in crystal cell numbers compared to the control. Not unexpectedly, reduced crystal cell numbers were observed in the cell cycle mutants *cdk1^E1-23^* and *cdk7^del^* as well as in the *sgg^M1-1^* and the *raf^12^* alleles, known to affect cellular growth and homeostasis [74,77,95,96,97,99,101]. However, larvae mutant for the other kinases developed far too many crystal cells (Figure 4). There was only one exception: the heterozygous *msn^102^*/+ mutant displayed nearly normal numbers, demonstrating the recessive character of the allele. In addition, blocking the activity of BubR1 and CamKII kinases, respectively, by inducing dominant negative forms in hemocytes, again caused a significant increase in crystal cell numbers (Figure 4). The downregulation of Akt1, CamKII and S6k caused an increase in crystal cell numbers similar to Pkc53E. We have shown previously that human PKCα, representing Pkc53E, phosphorylates Ser269 in a Su(H) peptide, while the three other human homologues piloted the neighboring Thr271 [47]. To date, we do not know whether Thr271 phosphorylation affects blood cell formation. However, all three kinases matched Pkc53E in their activity on crystal cell formation in support of this idea. Whether all the other kinases displaying similar phenotypes (i.e., Alc, Drak, Limk1, Mei-41, Msn, Par-1, Pkc98E, PKD, Slpr, Tkv and BubR1) also phosphorylate Su(H) or perhaps activate Pkc53E, Akt1, CamKII, S6k or upstream kinases, or whether they act independently in signaling pathways other than Notch, remains to be investigated.

**Figure 5:**
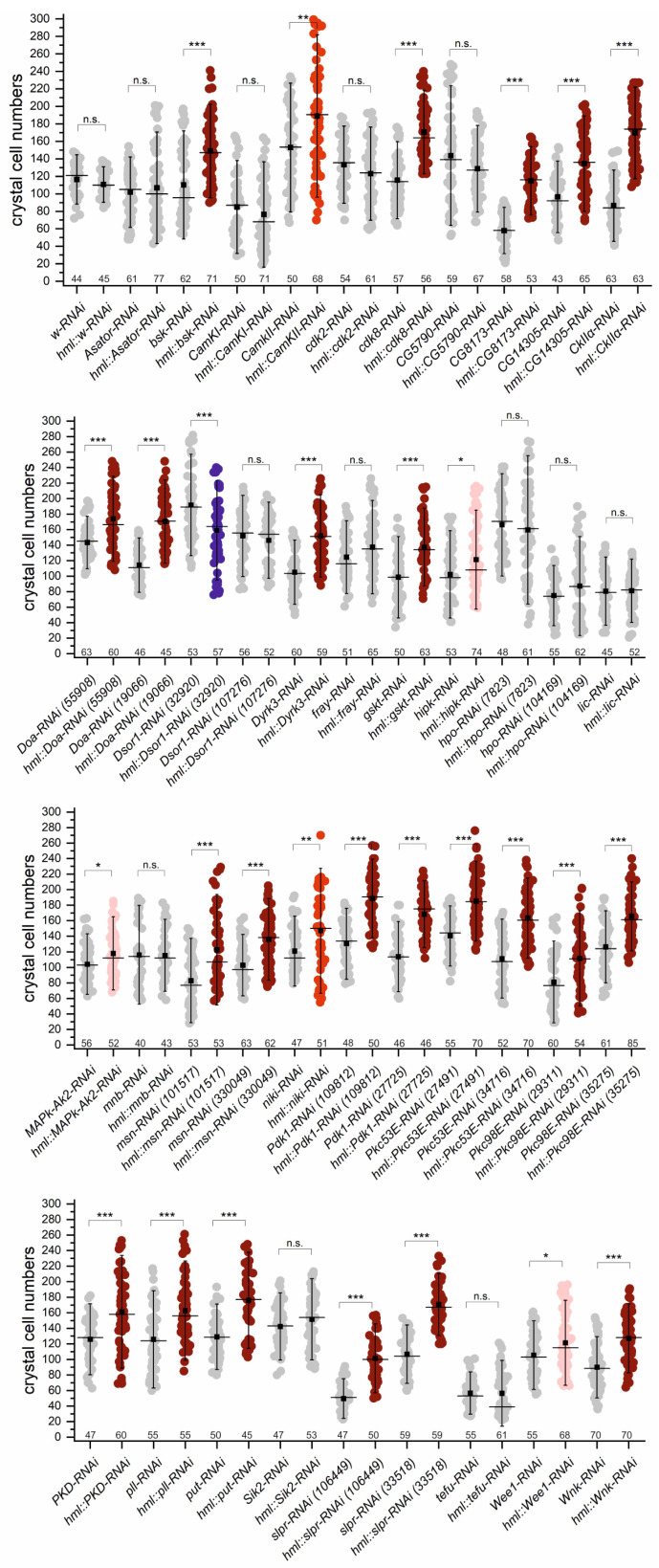
RNAi-mediated downregulation of Ser/Thr kinases affects crystal cell homeostasis. Numbers represent crystal cells in dorsal hematopoietic pockets from the last two segments in larvae of the indicated genotype (in alphabetical order). Every dot represents one larva (n, as shown above X-axis). Crystal cell increase is indicated in red and decrease in blue; coloration reflects significance. Whiskers, standard deviation; center line, median; center dot, average. For statistical analysis, ANOVA for multiple comparisons according to Dunnett’s test relative to control was employed (*** *p* ≤ 0.001, ** *p* ≤ 0.01, * *p* ≤ 0.05, and n.s. *p* > 0.05 not significant). The respective UAS-RNAi line served as control.

In order to confirm and extend the above result, we next employed a hemocyte-specific knockdown via RNAi, as a complete loss of a kinase activity may compromise development in general, or more specifically, mesoderm and/or hemocyte determination. To this end, we induced a total of 41 respective UAS-RNAi-lines with the HmlΔ-Gal4 driver covering an additional 27 Ser/Thr kinases (Figure 5). A complete list of the fly stocks used in these assays is presented in Table S1.

Confirming the above results, a knockdown of either kinase CamKII, Pkc53E, Pkc98E, PKD or Slpr, resulted in significantly higher crystal cell numbers. Moreover, a marked increase in crystal cell numbers was also observed, when either kinase Bsk, Cdk8, CG8173, CG14305, CKIIα, Doa, Dyrk3, Gskt, Msn, Niki, Pdk1, Pll, Put, or Wnk was downregulated. Albeit several of these kinases may play a role in JNK, TLR or WNT signaling, only Bsk and Msn as integral members of the JNK pathway, as well as Pll as central component of the TLR cascade, have ascribed roles during hematopoiesis [3,101,102,103]. Others like Pdk1 and Cdk8 are required in the broader context of apoptosis, proliferation and transcriptional regulation, or like Put for cardiac mesoderm development [3,56,66,77,79,104]. Notably, Pdk1 acts as a master kinase upstream of many kinases including Akt1, S6k and Pkc53E, easily explaining the observed phenotypes by kinase cross-talk [56,78,79]. Albeit it is conceivable that a network of cross-reacting kinases acts upstream of Pkc53E, hence indirectly regulating Su(H) phosphorylation, not all of them do. For example, whilst downregulation of Gskt induced crystal cell gain in the range of Pkc53E, the human homologue GSK3B was not able to phosphorylate a Su(H) peptide [47]. Instead, Gskt, similar to Wnk, may play a role in Wnt signaling, which regulates hemocyte precursor development [3,69,76,101]. Similarly, independent roles are expected for members of the JNK and TLR pathways, albeit cross-talk of signaling pathways during immune responses is well established [3,102,105].

In fact, 28 of the tested lines, representing 22 kinases, developed significantly more crystal cells when downregulated in hemocytes, i.e., nearly 70% of all tested lines, suggesting their involvement in blood cell homeostasis by restricting crystal cell numbers (Figure 5). In contrast, the knockdown of Dsor1 in hemocytes impaired crystal cell formation substantially (Figure 5). Apparently, Dsor1, the downstream kinase of Raf1 (MEK1), is critically required in hematopoiesis, in agreement with the results obtained for the *raf^12^* mutant (Figure 4) and earlier reports on the role of RTK signaling in hemocyte proliferation [96,105,106,107].

Tissue-specific induction of RNAi did not induce significantly altered crystal cell numbers in about one third of the crosses (Figure 5). In some cases, this may be due to ineffectiveness of the respective RNAi-line, e.g., for CamKI, Cdk2, CG5790 and Tefu [108]. UAS-RNAi lines affecting kinase activity of Asator, Fray, Hpo, Lic, Mnb and Sik2, however, have been reported before to be effective, suggesting that the respective kinases are not important for crystal cell development (Figure 5) [108,109].

**Figure 6:**
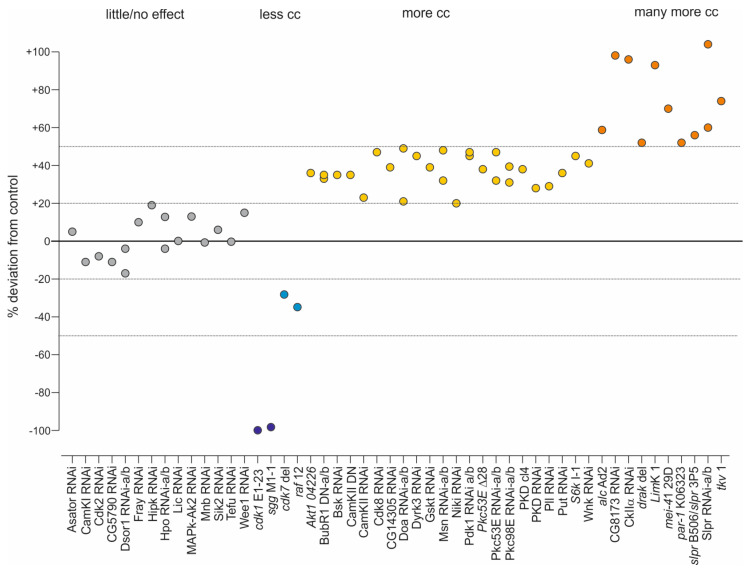
Effect of kinase loss on crystal cell formation (in the mutants and by hemocyte-specific knockdown, respectively). Crystal cell (cc) number increase/decrease depicted as percentage deviation from control, and sorted into five classes: no or little effect (+/−20% deviation, grey), strong loss (−50–100%, dark blue) and moderate loss (−20–50%, light blue), moderate increase (+20–50%, yellow) and strong increase (+50–100%, orange).

A combined analysis of the loss of function screens reveals that in sum 30 of 46 tested kinases, i.e., about 65%, appear to limit crystal cell formation, whereas only five promoted it. For an overview, kinases were sorted by their effect on crystal cell numbers into five classes with little effect (+/−20% deviation), moderate (up to 50% deviation) or strong increase or decrease (over 50%), respectively, when compared to the reference (Figure 6).

We observed some discrepancies with regard to the overexpression analyses. For example, RNAi-mediated downregulation of Asator or Sik2 did not affect crystal cell numbers, whereas the respective overexpression caused significant changes, perhaps reflecting inefficient RNA interference, or cross-talk of these kinases in the overexpression context. Similarly, whereas the downregulation of the kinases Alc, Cdk8 and CG8173 increased crystal cell numbers, no effect was seen in response to an overexpression, presumably due to inactivity of the respective kinase. Likewise, whereas crystal cell numbers were increased in the S6K mutant, neither ubiquitous nor hemocyte-specific expression of the activated S6K impaired crystal cell formation. Moreover, both the overexpression as well as the downregulation of either Dyrk3, Par-1 or Pdk1 caused a substantial increase in crystal cell numbers, suggesting a major impact in a regulatory network rather than a specific role in hematopoiesis.

## 4. Discussion

In this work, we have addressed the role of Ser/Thr kinases in larval crystal cell formation of *Drosophila*. In our search for the kinase specifically piloting Su(H) during hematopoiesis, we observed that the large majority of kinases we screened affected crystal cell formation. This result was unexpected, because our screens did not specifically aim at kinases known to be involved in the hematopoiesis of *Drosophila* but rather at those predicted to recognize Ser269 in the beta-trefoil domain of Su(H) [45,47]. Based on our results, most of these kinases have a role in restricting crystal cell development, whereas only a handful of kinases appeared to be required for crystal cell formation.

Previous screens applying RNA interference at large scale missed the apparently ubiquitous role of Ser/Thr kinases during hematopoiesis, which we attribute largely to the particular focus as well as the screening procedure. For example, one screen aimed at the identification of factors regulating the numbers of hemocytes deficient for the PDGF/VEGF receptor, coming up with components of the EcR pathway as suppressors and RTK signaling pathways as enhancers, including the downstream kinases, Akt1, S6K, Dsor and MAPK, respectively [106]. Indeed, the MAPK cascade, apart from regulating hemocyte proliferation, specifically inhibits IMD signaling in larval hemocytes, thereby preventing spurious immune activation and limiting the immune response [107]. In a recent large-scale screen, larval hemocytes were labelled with GFP, and the changes in fluorescent signal strength or distribution upon RNAi-mediated downregulation of gene activity was evaluated [110]. This screen again picked up Akt kinase’s relevance for hemocyte proliferation [110]. A specific contribution of Ser/Thr kinases to crystal cell formation, however, was not uncovered although the screening procedure included crystal cells as well. Presumably, more subtle changes went unnoticed. Taking into account that crystal cells normally make up just about 5% of the larval hemocytes, a doubling or halving of their numbers would not stand out in the bulk of plasmatocytes. Other recent RNAi screens addressed lamellocyte-based tumor formation without taking crystal cells into account [111,112]. Screening the *Drosophila* genome for tumor suppressors, only two Ser/Thr kinases with established roles in immunity were picked up, accounting for roughly three percent of the screened Ser/Thr kinases in total [111]. Likewise, any specific contributions of kinases to hemocyte differentiation or to specific immune responses were missed in the recent genome-wide analyses concentrating on the transcriptome rather than the proteome or the kinome [113,114,115].

**Figure 7:**
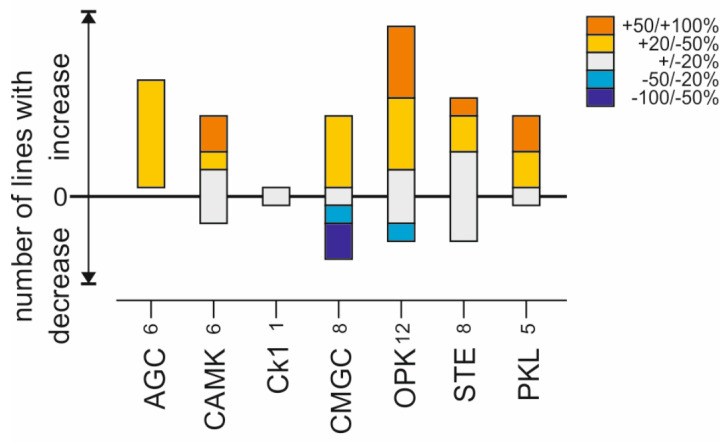
Influence of loss of kinase activity on crystal cell formation sorted by kinase class. Crystal cell number increase/decrease is depicted as percentage deviation from control. It was sorted into five classes: no or little effect (+/−20% deviation), moderate effect (+/−20–50%) and strong effect (+/−50–100%). Abbreviation of kinase classes: ACG, cAMP-/cGMP-dependent and protein kinase C; CAMK, Ca/calmodulin-dependent protein kinase; CKI, Casein-kinase I family; CMGC, Cdk, MAPK, GCK2 and CLK members; OPK, other protein kinases; STE, Ste7,11,20 gene homologues; PKL, protein kinase like. Numbers indicate represented genes.

Specifically aiming at crystal cell formation, our gain- and loss-of-function screens, however, uncovered an unexpected major contribution for Ser/Thr kinases. Nearly 75% of the investigated Ser/Thr kinases influenced blood cell homeostasis (35 of 46). Only the kinases Sgg, Cdk1, Cdk7, Raf and Dsor1 appeared to be required for the formation of crystal cells, since the respective mutant larvae, *sgg^M1-1^*, *cdk1^E1-23^*, *cdk7^del^* and *raf^12^*, as well as animals with a Dsor1 knockdown, lacked crystal cells, or developed significantly less than the controls (Figure 4 and Figure 5). A loss-of-function of the majority of the studied kinases, however, entailed a significant increase in crystal cell numbers. Interestingly, all kinase classes are represented, pointing to a much more complex regulation of blood cell homeostasis than anticipated so far (Figure 7). Hence, any class contains representatives that may be involved in blood cell homeostasis. However, members belonging to the AGC, the OPK and the PKL classes appear particularly important, since most of their mutants displayed increased crystal cell numbers. In contrast, CMGC members give a mixed picture, with roughly half-and-half support vs. restriction of crystal cell formation, in accord with this group comprising kinases with a rather general role in development and cellular homeostasis.

Quite intriguingly, our screens revealed an apparently general requirement of Ser/Thr kinases in blood cell homeostasis, and specifically in crystal cell formation. We did not prescreen kinase candidates for a likely involvement in hematopoiesis, but rather for their competence to phosphorylate Su(H) at Ser269, thereby assessing just about 20% of *Drosophila* Ser/Thr kinases. Hence, the group of kinases whose downregulation increases crystal cell numbers may be even larger. That all these kinases directly phosphorylate Su(H) is out of the question. Given the intricate network of kinase cross-reactivity it is conceivable that a fair number of them pilot a few kinases that either directly phosphorylate Su(H), like Pkc53E, or act upstream. Presumably, the majority of these kinases functions in signaling pathways unrelated to Su(H) activity. This may also explain why the downregulation of some kinases induced crystal cell numbers exceeding those caused by a loss of Pkc53E. For example, the rise in crystal cell numbers may reflect a general expansion of hemocytes or derive from the final differentiation of plasmatocytes into crystal cells. Excessive differentiation of plasmatocytes and crystal cells in response to JNK activation has been observed in the lymph gland [116], but neither in the hemolymph nor in the sessile compartment. In our hands, however, a knockdown of JNK activity caused an increase rather than a decrease in the numbers of sessile crystal cells. Crystal cells play various roles in innate immunity. Importantly, via the melanization cascade, they help in wound healing and combat bacterial and fungal infections by the production of cytotoxic reactive oxygen species (ROS) [3,102,117,118]. Apart from its function in redox signaling and oxidative stress, ROS is an important signaling molecule during *Drosophila* hematopoiesis [116,119,120]. Moreover, ample reports on redox-sensitivity of kinases and phosphatases point to a comprehensive cross-talk of several signaling pathways and the cellular redox signaling (reviewed in [119,120,121]). Redox-triggered mechanisms, cystein oxidation in particular, can alter the catalytic properties of kinases and phosphatases. Notably, redox regulation of protein tyrosine phosphatases and protein tyrosine kinases is well documented, and may alter the overall cellular phospho-status by also influencing Ser/Thr kinases. However, the activity of several Ser/Thr kinases is regulated by redox modifications as well. This has been demonstrated already for Akt1, MAPKs (e.g., JNK and p38), ATM, PKC and CAMKs (reviewed in: [119,121,122]). Clearly, signaling pathways regulating the cellular redox state are themselves subjected to redox regulation, arguing for an intensive cross-talk between the systems.

While our findings may reconcile the activity and cross-talk of the multitude of signaling pathways influencing blood cell development, proliferation and maintenance, we speculate that the innate immune responses of the *Drosophila* larva may in addition rely on the overall phospho-status within the larval blood cells. Hence, tinkering with kinases activities, e.g., by a hemocyte-specific knockdown, may lower the intracellular phospho-status, thereby promoting crystal cell differentiation and hence, increasing crystal cell numbers. This hypothesis could explain the rather indiscriminative response we observed. In this case, protein phosphorylation may act as a general measure for cellular stress, resulting in the activation of adaptive responses.

## 5. Conclusions

Blood cell homeostasis is under the influence of many signaling pathways granting proper immune responses to external stressors and infections. However, an unexpectedly large fraction of Ser/Thr kinases investigated in this work affected crystal cell formation, indicating their potential involvement beyond established hematopoietic signaling pathways. Some of these kinases may, directly or indirectly via cross-talk, cause the phosphorylation of Su(H) and thereby influence crystal cell numbers. Others, however, may be involved in blood cell formation through their activity in other signaling pathways unrelated to Notch signaling. Based on the rather indiscriminative response to the activity changes of Ser/Thr kinase in general, however, we speculate that the overall intracellular phospho-status in hemocytes serves as a fundamental measure for stress signals to adapt innate immune responses accordingly.

## Figures and Tables

**Table 1:** List of *Drosophila* Ser/Thr kinases assayed, and their human homologues.

*Drosophila* Kinase	Human Kinase	Class ^2^	*Drosophila* Kinase	Human Kinase	Class ^2^
Akt1 ^1^	AKT1/AKT2	*AGC*	Lic	MAP2K3	*CAMK*
Alc ^1^	PRKAB1/2	*PKL*	LimK1	Limk1/2	*OPK*
Asator ^1^	TTBK1	*CK1*	Mapk-Ak2	MAPKAPK3	*OPK*
Bsk	JNK2	*CMGC*	Mei-41	ATR	* PKL *
BubR1 ^1^	BUB1B	*OPK*	Mnb	DYRK1	*STE*
CaMKI	CAMK1D	*CAMK*	Msn	MAP4K4	*STE*
CaMKII ^1^	CAMK2A/2D	*CAMK*	Niki	NEK3/9	*CAMK*
Cdk1	CDK1	*CMGC*	Par-1 ^1^	MAPK3	* AGC *
Cdk2 ^1^	CDK2	*CMGC*	Pdk1 ^1^	PDK1	* AGC *
Cdk7 ^1^	CDK7	*CMGC*	Pkc53E ^1^	PRKCA	*AGC*
Cdk8 ^1^	CDK8	*CMGC*	Pkc98E	PRKCE	* AGC *
CKIIa	CK2-alpha1/2	*OPK*	PKD	PRKD1	* AGC *
CG5790	CDC7/ASK	*STE*	Pll	IRAK4	* PKL *
CG8173 ^1^	PBK	*OPK*	Put	ACV-R2A	* PKL *
CG14305/Tssk	TSSK1	*OPK*	Raf ^1^	RAF1/B-RAF	* OPK *
Doa	CLK2	*CMGC*	S6K ^1^	RPS6KB1	* AGC *
Dsor1	MEK1	*STE*	Sgg	GSK3A	* CMGC *
Drak	STK17B	*CAMK*	Sik2 ^1^	SIK2	* CAMK *
Dyrk3 ^1^	DYRK2	*OPK*	Slpr	MAP3K1/9	* STE *
Fray ^1^	STK39	*STE*	Tefu	ATM	* PKL *
Gskt ^1^	GSK3B	*CMGC*	Tkv ^1^	BMP-R1A	* OPK *
HipK ^1^	HIPK	*OPK*	Wee1 ^1^	WEE1	* OPK *
Hpo	STK3	*STE*	Wnk ^1^	WNK1/2	* OPK *

^1^ Kinases used in both, gain and loss-of function assays. ^2^ Abbreviation of classes as in Figure 2.

## Data Availability

The data presented in this study are available in the article and the Supplementary Materials.

## Supplementary Materials

The following supporting information can be downloaded at: https://www.mdpi.com/article/10.3390/cells13070576/s1, Table S1: List of fly strains; Table S2: List of oligonucleotides used for constructs.

## References

[1] Gold K.S., Brückner K. (2014). *Drosophila* as a model for the two myeloid blood cell systems in vertebrates. Exp. Hematol..

[2] Letourneau M., Lapraz F., Sharma A., Vanzo N., Waltzer L., Crozatier M. (2016). *Drosophila* hematopoiesis under normal conditions and in response to immune stress. FEBS Lett..

[3] Banerjee U., Girard J.R., Goins L.M., Spratford C.M. (2019). *Drosophila* as a genetic model for hematopoiesis. Genetics.

[4] Csordás G., Gábor E., Honti V. (2021). There and back again: The mechanisms of differentiation and transdifferentiation in *Drosophila* blood cells. Dev. Biol..

[5] Makhijani K., Brückner K. (2012). Of blood cells and the nervous system: Hematopoiesis in the *Drosophila* larva. Fly.

[6] Makhijani K., Alexander B., Tanaka T., Rulifson E., Brückner K. (2011). The peripheral nervous system supports blood cell homing and survival in the *Drosophila* larva. Development.

[7] Makhijani K., Alexander B., Rao D., Petraki S., Herboso L., Kukar K., Batool I., Wachner S., Gold K.S., Wong C. (2017). Regulation of *Drosophila* hematopoietic sites by Activin-β from active sensory neurons. Nat. Commun..

[8] Márkus R., Laurinyecz B., Kurucz E., Honti V., Bajusz I., Sipos B., Somogyi K., Kronhamn J., Hultmark D., Andó I. (2009). Sessile hemocytes as a hematopoietic compartment in *Drosophila melanogaster*. Proc. Natl. Acad. Sci. USA.

[9] Leitão A.B., Sucena É. (2015). *Drosophila* sessile hemocyte clusters are true hematopoietic tissues that regulate larval blood cell differentiation. Elife.

[10] Honti V., Csordás G., Márkus R., Kurucz E., Jankovics F., Andó I. (2010). Cell lineage tracing reveals the plasticity of the hemocyte lineages and of the hematopoietic compartments in *Drosophila melanogaster*. Mol. Immunol..

[11] Stofanko M., Kwon S.Y., Badenhorst P. (2010). Lineage tracing of lamellocytes demonstrates *Drosophila* macrophage plasticity. PLoS ONE.

[12] Anderl I., Vesala L., Ihalainen T.O., Vanha-Aho L.M., Andó I., Rämet M., Hultmark D. (2016). Transdifferentiation and Proliferation in Two Distinct Hemocyte Lineages in *Drosophila melanogaster* Larvae after Wasp Infection. PLoS Pathog..

[13] Bernardoni R., Vivancos V., Giangrande A. (1997). Glide/gcm is expressed and required in the scavenger cell lineage. Dev. Biol..

[14] Alfonso T.B., Jones B.W. (2002). *gcm2* promotes glial cell differentiation and is required with glial cells missing for macrophage development in *Drosophila*. Dev. Biol..

[15] Cattenoz P.B., Popkova A., Southall T.D., Aiello G., Brand A.H., Giangrande A. (2016). Functional conservation of the Glide/Gcm regulatory network controlling glia, hemocyte, and tendon cell differentiation in *Drosophila*. Genetics.

[16] Lebestky T., Chang T., Hartenstein V., Banerjee U. (2000). Specification of *Drosophila* hematopoietic lineage by conserved transcription factors. Science.

[17] Brückner K., Kockel L., Duchek P., Luque C.M., Rørth P., Perrimon N. (2004). The PDGF/VEGF receptor controls blood cell survival in *Drosophila*. Dev. Cell..

[18] Pastor-Pareja J.C., Wu M., Xu T. (2008). An innate immune response of blood cells to tumors and tissue damage in *Drosophila*. Dis. Model Mech..

[19] Mondal B.C., Mukherjee T., Mandal L., Evans C.J., Sinenko S.A., Martinez-Agosto J.A., Banerjee U. (2011). Interaction between differentiating cell- and niche-derived signals in hematopoietic progenitor maintenance. Cell.

[20] Benmimoun B., Polesello C., Waltzer L., Haenlin M. (2012). Dual role for Insulin/TOR signaling in the control of hematopoietic progenitor maintenance in *Drosophila*. Development.

[21] Dragojlovic-Munther M., Martinez-Agosto J.A. (2012). Multifaceted roles of PTEN and TSC orchestrate growth and differentiation of *Drosophila* blood progenitors. Development.

[22] Shim J., Mukherjee T., Banerjee U. (2012). Direct sensing of systemic and nutritional signals by haematopoietic progenitors in *Drosophila*. Nat. Cell Biol..

[23] Parsons B., Foley E. (2013). The *Drosophila* platelet-derived growth factor and vascular endothelial growth factor-receptor related (Pvr) protein ligands Pvf2 and Pvf3 control hemocyte viability and invasive migration. J. Biol. Chem..

[24] Razzell W., Evans I.R., Martin P., Wood W. (2013). Calcium flashes orchestrate the wound inflammatory response through DUOX activation and hydrogen peroxide release. Curr. Biol..

[25] Ferguson G.B., Martinez-Agosto J.A. (2017). The TEAD family transcription factor Scalloped regulates blood progenitor maintenance and proliferation in *Drosophila* through PDGF/VEGFR receptor (Pvr) signaling. Dev. Biol..

[26] Cho B., Spratford C.M., Yoon S., Cha N., Banerjee U., Shim J. (2018). Systemic control of immune cell development by integrated carbon dioxide and hypoxia chemosensation in *Drosophila*. Nat. Commun..

[27] Bakopoulos D., Beadle L.F., Esposito K.M., Mirth C.K., Warr C.G., Johnson T.K. (2020). Insulin-like signalling influences the coordination of larval hemocyte number with body size in *Drosophila melanogaster*. G3.

[28] Evans C.J., Liu T., Girard J.R., Banerjee U. (2022). Injury-induced inflammatory signaling and hematopoiesis in *Drosophila*. Proc. Natl. Acad. Sci. USA.

[29] Fossett N., Tevosian S.G., Gajewski K., Zhang Q., Orkin S.H., Schulz R.A. (2001). The Friend of GATA proteins U-shaped, FOG-1, and FOG-2 function as negative regulators of blood, heart, and eye development in Drosophila. Proc. Natl. Acad. Sci. USA.

[30] Ferjoux G., Auge B., Boyer K., Haenlin M., Waltzer L. (2007). A GATA/RUNX cis-regulatory module couples *Drosophila* blood cell commitment and differentiation into crystal cells. Dev. Biol..

[31] Waltzer L., Bataillé L., Peyrefitte S., Haenlin M. (2002). Two isoforms of Serpent containing either one or two GATA zinc fingers have different roles in *Drosophila* haematopoiesis [published correction appears in *EMBO J.*] **2016**, *35*, 553. EMBO J..

[32] Waltzer L., Ferjoux G., Bataillé L., Haenlin M. (2003). Cooperation between the GATA and RUNX factors Serpent and Lozenge during *Drosophila* hematopoiesis. EMBO J..

[33] Lebestky T., Jung S.-H., Banerjee U. (2003). A Serrate-expressing signaling center controls *Drosophila* hematopoiesis. Genes Dev..

[34] Mukherjee T., Kim W.S., Mandal L., Banerjee U. (2011). Interaction between Notch and Hif-alpha in development and survival of *Drosophila* blood cells. Science.

[35] Terriente-Felix A., Li J., Collins S., Mulligan A., Reekie I., Bernard F., Krejci A., Bray S. (2013). Notch cooperates with Lozenge/Runx to lock haemocytes into a differentiation programme. Development.

[36] Frankenreiter L., Gahr B.M., Schmid H., Zimmermann M., Deichsel S., Hoffmeister P., Turkiewicz A., Borggrefe T., Oswald F., Nagel A.C. (2021). Phospho-site mutations in transcription factor Suppressor of Hairless impact Notch signaling activity during hematopoiesis in *Drosophila*. Front. Cell Dev. Biol..

[37] Bray S.J. (2006). Notch signalling: A simple pathway becomes complex. Nat. Rev. Mol. Biol..

[38] Bray S.J. (2016). Notch signalling in context. Nat. Rev. Mol. Biol..

[39] Bray S., Furriols M. (2001). Notch pathway: Making sense of Suppressor of Hairless. Curr. Biol..

[40] Hori K., Sen A., Artavanis-Tsakonas S. (2013). Notch signaling at a glance. J. Cell Sci..

[41] Kopan R., Ilagan M.X. (2009). The canonical Notch signaling pathway: Unfolding the activation mechanism. Cell.

[42] Kovall R.A., Blacklow S.C. (2010). Mechanistic insights into Notch receptor signaling from structural and biochemical studies. Curr. Top. Dev. Biol..

[43] Kovall R.A., Gebelein B., Sprinzak D., Kopan R. (2017). The canonical Notch signaling pathway: Structural and biochemical insights into shape, sugar, and force. Dev. Cell..

[44] Giaimo B.D., Gagliani E.K., Kovall R.A., Borggrefe T. (2021). Transcription Factor RBPJ as a Molecular Switch in Regulating the Notch Response. Adv. Exp. Med. Biol..

[45] Nagel A.C., Auer J.S., Schulz A., Pfannstiel J., Yuan Z., Collins C.E., Kovall R.A., Preiss A. (2017). Phosphorylation of Suppressor of Hairless impedes its DNA-binding activity. Sci. Rep..

[46] Cherbas L., Willingham A., Zhang D., Yang L., Zou Y., Eads B.D., Carlson J.W., Landolin J.M., Kapranov P., Dumais J. (2011). The transcriptional diversity of 25 *Drosophila* cell lines. Genome Res..

[47] Deichsel S., Frankenreiter L., Fechner J., Gahr B.M., Zimmermann M., Mastel H., Preis I., Preiss A., Nagel A.C. (2023). Inhibition of Notch activity by phosphorylation of CSL in response to parasitization in Drosophila. eLife.

[48] Rizki M.T.M. (1957). Alterations in the haemocyte population of *Drosophila melanogaster*. J. Morphol..

[49] Lanot R., Zachary D., Holder F., Meister M. (2001). Postembryonic Hematopoiesis in *Drosophila*. Dev. Biol..

[50] Brand A.H., Perrimon N. (1993). Targeted gene expression as a means of altering cell fates and generating dominant phenotypes. Development.

[51] Wodarz A., Hinz U., Engelbert M., Knust E. (1995). Expression of *crumbs* confers apical character on plasma membrane domains of ectodermal epithelia of *Drosophila*. Cell.

[52] Bischof J., Björklund M., Furger E., Schertel C., Taipale J., Basler K. (2013). A versatile platform for creating a comprehensive UAS-ORFeome library in *Drosophila*. Development.

[53] Spasić M.R., Callaerts P., Norga K.K. (2008). *Drosophila alicorn* is a neuronal maintenance factor protecting against activity-induced retinal degeneration. J. Neurosci..

[54] Swarup S., Verheyen E.M. (2011). *Drosophila* homeodomain-interacting protein kinase inhibits the Skp1-Cul1-F-box E3 ligase complex to dually promote Wingless and Hedgehog signaling. Proc. Natl. Acad. Sci. USA.

[55] Vaccari T., Rabouille C., Ephrussi A. (2005). The *Drosophila* PAR-1 spacer domain is required for lateral membrane association and for polarization of follicular epithelial cells. Curr. Biol..

[56] Rintelen F., Stocker H., Thomas G., Hafen E. (2001). PDK1 regulates growth through Akt and S6K in *Drosophila*. Proc. Natl. Acad. Sci. USA.

[57] Wehr M.C., Holder M.V., Gailite I., Saunders R.E., Maile T.M., Ciirdaeva E., Instrell R., Jiang M., Howell M., Rossner M.J. (2013). Salt-inducible kinases regulate growth through the Hippo signalling pathway in *Drosophila*. Nat. Cell Biol..

[58] Bischof J., Maeda R.K., Hediger M., Karch F., Basler K. (2007). An optimized transgenesis system for *Drosophila* using germ-line-specific phiC31 integrases. Proc. Natl. Acad. Sci. USA.

[59] Neubueser D., Hipfner D.R. (2010). Overlapping roles of *Drosophila* Drak and Rok kinases in epithelial tissue morphogenesis. Mol. Biol. Cell.

[60] Schindelin J., Arganda-Carreras I., Frise E., Kaynig V., Longair M., Pietzsch T., Preibisch S., Rueden C., Saalfeld S., Schmid B. (2012). Fiji: An open-source platform for biological-image analysis. Nat. Methods.

[61] Xue Y., Liu Z., Cao J., Ma Q., Gao X., Wang Q., Jin C., Zhou Y., Wen L., Ren J. (2011). GPS 2.1: Enhanced prediction of kinase-specific phosphorylation sites with an algorithm of motif length selection. Protein. Eng. Des. Sel..

[62] Morrison D.K., Murakami M.S., Cleghon V. (2000). Protein kinases and phosphatases in the *Drosophila* genome. J. Cell Biol..

[63] Manning G., Whyte D.B., Martinez R., Hunter T., Sudarsanam S. (2002). The protein kinase complement of the human genome. Science.

[64] Landry C.R., Levy E.D., Michnick S.W. (2009). Weak functional constraints on phosphoproteomes. Trends Genet..

[65] Gnad F., Forner F., Zielinska D.F., Birney E., Gunawardena J., Mann M. (2010). Evolutionary constraints of phosphorylation in eukaryotes, prokaryotes, and mitochondria. Mol. Cell. Proteom..

[66] Sopko R., Foos M., Vinayagam A., Zhai B., Binari R., Hu Y., Randklev S., Perkins L.A., Gygi S.P., Perrimon N. (2014). Combining genetic perturbations and proteomics to examine kinase-phosphatase networks in *Drosophila* embryos. Dev. Cell..

[67] Johnson J.L., Yaron T.M., Huntsman E.M., Kerelsky A., Song J., Regev A., Lin T.Y., Liberatore K., Cizin D.M., Cohen B.M. (2023). An atlas of substrate specificities for the human serine/threonine kinome. Nature.

[68] Rahmani Z., Gagou M.E., Lefebvre C., Emre D., Karess R.E. (2009). Separating the spindle, checkpoint, and timer functions of BubR1. J. Cell. Biol..

[69] Kalamegham R., Sturgill D., Siegfried E., Oliver B. (2007). *Drosophila mojoless*, a retroposed GSK-3, has functionally diverged to acquire an essential role in male fertility. Mol. Biol. Evol..

[70] Şahin H.B., Sayın S., Holder M., Buğra K., Çelik A. (2020). Salt inducible kinases as novel Notch interactors in the developing *Drosophila* retina. PLoS ONE.

[71] Larochelle S., Pandur J., Fisher R.P., Salz H.K., Suter B. (1998). Cdk7 is essential for mitosis and for in vivo Cdk-activating kinase activity. Genes Dev..

[72] Weinkove D., Leevers S.J. (2000). The genetic control of organ growth: Insights from *Drosophila*. Curr. Opin. Genet. Dev..

[73] Stocker H., Hafen E. (2000). Genetic control of cell size. Curr. Opin. Genet. Dev..

[74] Tapon N., Moberg K.H., Hariharan I.K. (2001). The coupling of cell growth to the cell cycle. Curr. Opin. Cell Biol..

[75] Saucedo L.J., Edgar B.A. (2002). Why size matters: Altering cell size. Curr. Opin. Genet. Dev..

[76] Serysheva E., Berhane H., Grumolato L., Demir K., Balmer S., Bodak M., Boutros M., Aaronson S., Mlodzik M., Jenny A. (2013). Wnk kinases are positive regulators of canonical Wnt/β-catenin signalling [published correction appears in *EMBO Rep*.] **2013**, *14*, 845. EMBO Rep..

[77] Cho Y.S., Li S., Wang X., Zhu J., Zhuo S., Han Y., Yue T., Yang Y., Jiang J. (2020). CDK7 regulates organ size and tumor growth by safeguarding the Hippo pathway effector Yki/Yap/Taz in the nucleus. Genes Dev..

[78] Cho K.S., Lee J.H., Kim S., Kim D., Koh H., Lee J., Kim C., Kim J., Chung J. (2001). *Drosophila* phosphoinositide-dependent kinase-1 regulates apoptosis and growth via the phosphoinositide 3-kinase-dependent signaling pathway. Proc. Natl. Acad. Sci. USA.

[79] Roth S.W., Bitterman M.D., Birnbaum M.J., Bland M.L. (2018). Innate immune signaling in *Drosophila* blocks insulin signaling by uncoupling PI(3,4,5)P3 production and Akt activation. Cell Rep..

[80] Hariharan I.K., Bilder D. (2006). Regulation of imaginal disc growth by tumor-suppressor genes in *Drosophila*. Annu. Rev. Genet..

[81] Huang H.L., Wang S., Yin M.X., Dong L., Wang C., Wu W., Lu Y., Feng M., Dai C., Guo X. (2013). Par-1 regulates tissue growth by influencing hippo phosphorylation status and hippo-salvador association. PLoS Biol..

[82] Richardson H.E., Portela M. (2017). Tissue growth and tumorigenesis in *Drosophila*: Cell polarity and the Hippo pathway. Curr. Opin. Cell. Biol..

[83] Qi H., Yao C., Cai W., Girton J., Johansen K.M., Johansen J. (2009). Asator, a tau-tubulin kinase homolog in *Drosophila* localizes to the mitotic spindle. Dev. Dyn..

[84] Gwack Y., Sharma S., Nardone J., Tanasa B., Iuga A., Srikanth S., Okamura H., Bolton D., Feske S., Hogan P.G. (2006). A genome-wide *Drosophila* RNAi screen identifies DYRK-family kinases as regulators of NFAT. Nature.

[85] Destalminil-Letourneau M., Morin-Poulard I., Tian Y., Vanzo N., Crozatier M. (2021). The vascular niche controls *Drosophila* hematopoiesis via fibroblast growth factor signaling. Elife.

[86] Neumann C., Cohen S. (1997). Morphogens and pattern formation. Bioessays.

[87] Vincent J.P., Briscoe J. (2001). Morphogens. Curr. Biol..

[88] Bier E., De Robertis E.M. (2015). BMP gradients: A paradigm for morphogen-mediated developmental patterning. Science.

[89] Maggert K., Levine M., Frasch M. (1995). The somatic-visceral subdivision of the embryonic mesoderm is initiated by dorsal gradient thresholds in *Drosophila*. Development.

[90] Rusch J., Levine M. (1996). Threshold responses to the dorsal regulatory gradient and the subdivision of primary tissue territories in the *Drosophila* embryo. Curr. Opin. Genet. Dev..

[91] Duronio R.J. (1999). Establishing links between developmental signaling pathways and cell-cycle regulation in *Drosophila*. Curr. Opin. Genet. Dev..

[92] Restrepo S., Zartman J.J., Basler K. (2014). Coordination of patterning and growth by the morphogen DPP. Curr. Biol..

[93] Brand A.H., Perrimon N. (1994). Raf acts downstream of the EGF receptor to determine dorsoventral polarity during *Drosophila* oogenesis. Genes Dev..

[94] Luo H., Rose P.E., Roberts T.M., Dearolf C.R. (2002). The Hopscotch Jak kinase requires the Raf pathway to promote blood cell activation and differentiation in *Drosophila*. Mol. Genet. Genomics..

[95] Shilo B.Z. (2014). The regulation and functions of MAPK pathways in *Drosophila*. Methods.

[96] Hayashi S., Ogura Y. (2020). ERK signaling dynamics in the morphogenesis and homeostasis of *Drosophila*. Curr. Opin. Genet. Dev..

[97] Lee L.A., Orr-Weaver T.L. (2003). Regulation of cell cycles in *Drosophila* development: Intrinsic and extrinsic cues. Annu Rev Genet..

[98] Stumpff J., Duncan T., Homola E., Campbell S.D., Su T.T. (2004). *Drosophila* Wee1 kinase regulates Cdk1 and mitotic entry during embryogenesis. Curr. Biol..

[99] Brantley S.E., Di Talia S. (2021). Cell cycle control during early embryogenesis. Development.

[100] Cheng H.C., Matsuura I., Wang J.H. (1993). In vitro substrate specificity of protein tyrosine kinases. Mol. Cell. Biochem..

[101] Sinenko S.A., Mandal L., Martinez-Agosto J.A., Banerjee U. (2009). Dual role of wingless signaling in stem-like hematopoietic precursor maintenance in *Drosophila*. Dev. Cell..

[102] Bidla G., Dushay M.S., Theopold U. (2007). Crystal cell rupture after injury in *Drosophila* requires the JNK pathway, small GTPases and the TNF homolog Eiger. J. Cell Sci..

[103] Hultmark D., Andó I. (2022). Hematopoietic plasticity mapped in *Drosophila* and other insects. eLife.

[104] Gobert V., Osman D., Bras S., Augé B., Boube M., Bourbon H.M., Horn T., Boutros M., Haenlin M., Waltzer L. (2010). A genome-wide RNA interference screen identifies a differential role of the mediator CDK8 module subunits for GATA/ RUNX-activated transcription in *Drosophila*. Mol. Cell Biol..

[105] Sopko R., Lin Y.B., Makhijani K., Alexander B., Perrimon N., Brückner K. (2015). A systems-level interrogation identifies regulators of *Drosophila* blood cell number and survival. PLoS Genet..

[106] Karim F.D., Chang H.C., Therrien M., Wassarman D.A., Laverty T., Rubin G.M. (1996). A screen for genes that function downstream of Ras1 during *Drosophila* eye development. Genetics.

[107] Ragab A., Buechling T., Gesellchen V., Spirohn K., Boettcher A.L., Boutros M. (2011). *Drosophila* Ras/MAPK signalling regulates innate immune responses in immune and intestinal stem cells. EMBO J..

[108] Perkins L.A., Holderbaum L., Tao R., Hu Y., Sopko R., McCall K., Yang-Zhou D., Flockhart I., Binari R., Shim H.S. (2015). The Transgenic RNAi Project at Harvard Medical School: Resources and Validation. Genetics.

[109] Dietzl G., Chen D., Schnorrer F., Su K.C., Barinova Y., Fellner M., Gasser B., Kinsey K., Oppel S., Scheiblauer S. (2007). A genome-wide transgenic RNAi library for conditional gene inactivation in *Drosophila*. Nature.

[110] Evans C.J., Olson J.M., Mondal B.C., Kandimalla P., Abbasi A., Abdusamad M.M., Acosta O., Ainsworth J.A., Akram H.M., Albert R.B. (2021). A functional genomics screen identifying blood cell development genes in *Drosophila* by undergraduates participating in a course-based research experience. G3.

[111] Avet-Rochex A., Boyer K., Polesello C., Gobert V., Osman D., Roch F., Augé B., Zanet J., Haenlin M., Waltzer L. (2010). An in vivo RNA interference screen identifies gene networks controlling *Drosophila melanogaster* blood cell homeostasis. BMC Dev. Biol..

[112] Järvelä-Stölting M., Vesala L., Maasdorp M.K., Ciantar J., Rämet M., Valanne S. (2021). Proteasome α6 subunit negatively regulates the JAK/STAT pathway and blood cell activation in *Drosophila melanogaster*. Front. Immunol..

[113] Cho B., Yoon S.-H., Lee D., Koranteng F., Tattikota S.G., Cha N., Shin M., Do H., Hu Y., Oh S.Y. (2020). Single-cell transcriptome maps of myeloid blood cell lineages in *Drosophila*. Nat. Commun..

[114] Tattikota S.G., Cho B., Liu Y., Hu Y., Barrera V., Steinbaugh M.J., Yoon S.H., Comjean A., Li F., Dervis F. (2020). A single-cell survey of *Drosophila* blood. eLife.

[115] Girard J.R., Goins L.M., Vuu D.M., Sharpley M.S., Spratford C.M., Mantri S.R., Banerjee U. (2021). Paths and pathways that generate cell-type heterogeneity and developmental progression in hematopoiesis. eLife.

[116] Owusu-Ansah E., Banerjee U. (2009). Reactive oxygen species prime *Drosophila* haematopoietic progenitors for differentiation. Nature.

[117] Myers A.L., Harris C.M., Choe K.M., Brennan C.A. (2018). Inflammatory production of reactive oxygen species by *Drosophila* hemocytes activates cellular immune defenses. Biochem. Biophys. Res. Commun..

[118] Dziedziech A., Theopold U. (2022). Proto-Pyroptosis: An ancestral origin for mammalian inflammatory cell death mechanism in *Drosophila melanogaster*. J. Mol. Biol..

[119] Kamata H., Hirata H. (1999). Redox regulation of cellular signalling. Cell. Signal..

[120] Schieber M., Chandel N.S. (2014). ROS function in redox signaling and oxidative stress. Curr. Biol..

[121] Corcoran A., Cotter T.G. (2013). Redox regulation of protein kinases. Febs. J..

[122] Takata T., Araki S., Tsuchiya Y., Watanabe Y. (2020). Oxidative stress orchestrates MAPK and nitric-oxide synthase signal. Int. J. Mol. Sci..

