# Supplementary material for: Numerous Serine/Threonine Kinases Affect Blood Cell Homeostasis in Drosophila melanogaster
Source: Cells. 2024 Mar 26;13(7):576. doi: 10.3390/cells13070576 (PMC11011202; doi:10.3390/cells13070576)
Supplement: Supplementary file 1 [file cells-13-00576-s001.zip › cells-2903637-supplementary.pdf]

Supplemental Table S1

List of fly strains

Drosophila melanogaster, general stocks		
Abbreviation	**Genotype**	**Identifier/Reference**
Su(H)^{gwt}	*y*^{1} *w*^{67c23}*; Su(H)*^{gwt}	[36]
da-Gal4	*w[*]; P{w[+mW.hs]= Gal4-da.G32}2; P{w[+mW.hs]= Gal4-da.G32}UH1*	*BDSC 55849*
HmlΔ-Gal4; hml-Gal4	*w1118; P{w+mC=Hml-GAL4.Delta}3*	BDSC 30141
UAS-*white-RNAi*	*y1 v1; P{y+t7.7 v+t1.8=TRiP.JF01574}attP2*	BDSC 31231
UAS-lacZ	*w[*]; P{w[+mC]=UAS-lacZ.Exel}2*	BDSC 8529
vasa-φC31; 96E-attB/TM3	*vasa-φC31; 96E-attB*/TM3	[58]
Drosophila melanogaster stocks used for overexpression		
Abbreviation	**Genotype**	**Identifier/Reference**
Akt1	*UAS-myrAkt1/ TM6*	BDSC 50758
Alc	*UAS-alicorn/ TM6*	[53]
Asator	*EP-Asator/ In(4)Ci*^{D}	BDSC 44664
BubR1	*UAS-BubR1 3xHA/ TM6*	FO F001047
CamKII act	*UAS-CamKII*^{act} *T287D*	BDSC 29665
Cdk2	*UAS-Cdk2myc*	BDSC 6634
Cdk7	*UASt-attB-HA-Cdk7*	This work
Cdk8	*UAS-Cdk8 3xHA*	FO F001713
CG8173 = PBK	*EP-CG8173*	BDSC 21876
Dyrk3	*UASt-attB HA-Dyrk3*	This work
Fray	*UASt-attB-HA-Frayed*	This work
Gskt	*UASt-attB-HA-Gasket*	This work
HipK	*UAS-HipK2 HA*	[54]
Par-1	*TV3 UASg-eGFP-Par-1 (N1S)*$^{\#75}$*/TM2*	[55]
Pdk1	*yw; UAS-dPDK1*^{A467V}	[56]
Pkc53E	*UASt-attB-HA-Pkc53E*	[47]
Pkc53E^{EDDD}	*UASt-attB-HA-Pkc53E*^{EDDD}	[47]
Raf	*UAS-Raf*	BDSC 2033
S6K act	*UAS-S6K*^{T398E}	BDSC 6912
Sik2 act	*UAS-SIK2*^{S1032A}*/ SM6B*	[57]
Tkv act	*UAS-tkv*^{CA} *3rd*	BDSC 36537
Wee1	*UAS-Wee1*	FO F001334
Wnk	*EP-Wnk [EY10165]/TM6*	BDSC 16970
Drosophila melanogaster stocks used in the loss of function screens		
Abbreviation	**Genotype**	**Identifier/Reference**
Akt1	*ry506P{ry+t7.2=PZ}Akt 04226/TM6B Tb1 ubi-GFP*	BDSC 11627
Alc	*alcAd2/CyO-GFP*	BDSC 5510
Asator	*y1 v1; P{y+t7.7 v+t1.8=TRiP.HMC04184}attP2*	BDSC 55902
Bsk	*y1 sc* v1 sev21; P{y+t7.7 v+t1.8=TRiP.HMC03539}attP2*	BDSC 53310
BubR1	*y1 w1118; P{w+mC=UAS-BubR1.DN}2*	BDSC 8380
BubR1	*y1 w1118; P{w+mC=UAS-BubR1.DN}3*	BDSC 8382
CamKI	*y1 v1; P{y+t7.7 v+t1.8=TRiP.JF02268}attP2*	BDSC 26726

CamKII	*y1 sc* v1 sev21; P{y+t7.7 v+t1.8=TRiP.GL00237}attP2/TM6B*	BDSC 35330
CamKII	*w*; P{w+mC=UAS-CaMKII.T287A}3B3*	BDSC 29663
Cdk1	*Cdk1E1-23 b1 pr1 cn1/CyO-GFP*	BDSC 6629
Cdk2	*y1 v1; P{y+t7.7 v+t1.8=TRiP.HM05163}attP2*	BDSC 28952
Cdk7	*Df(1)JB254, P{w+mC=snf+,dhd+}S SL2, w*/FM7-GFP*	BDSC 4557
Cdk8	*y1 sc* v1 sev21; P{y+t7.7 v+t1.8=TRiP.HMS05476}attP40*	BDSC 67010
CG5790	*y1 v1; P{y+t7.7 v+t1.8=TRiP.HMJ23933}attP40/CyO-GFP*	BDSC 62453
CG8173	*y1 v1; P{y+t7.7 v+t1.8=TRiP.JF01161}attP2*	BDSC 31586
CG14305	*y1 sc* v1 sev21; P{y+t7.7 v+t1.8=TRiP.HMC05158}attP40*	BDSC 62151
CkII α	*y1 v1; P{y+t7.7 v+t1.8=TRiP.JF01436}attP2*	BDSC 31645
Doa	*y1 v1; P{y+t7.7 v+t1.8=TRiP.HMC04193}attP2*	BDSC 55908
Doa	*w1118; P{GD8588}v19066*	VDRC 19066
Drak	*Drak*^{del}	[59]
Dsor1	*y1 sc* v1 sev21; P{y+t7.7 v+t1.8=TRiP.HMS00710}attP2/TM3, Sb1*	BDSC 32920
Dsor1	*P{KK102276}VIE-260B*	VDRC 107276
Dyrk3	*y1 sc* v1 sev21; P{y+t7.7 v+t1.8=TRiP.HMC04155}attP2*	BDSC 55882
Fray	*y1 sc* v1 sev21; P{y+t7.7 v+t1.8=TRiP.HMS01794}attP2*	BDSC 38327
Gskt	*y1 sc* v1 sev21; P{y+t7.7 v+t1.8=TRiP.HMC05795}attP2*	BDSC 64922
Hipk	*y1 sc* v1 sev21; P{y+t7.7 v+t1.8=TRiP.HMC05078}attP40*	BDSC 60084
Hpo	*w1118; P{GD1570}v7823*	VDRC 7823
Hpo	*P{KK101704}VIE-260B*	VDRC 104169
Lic	*w1118; P{GD7546}v20166/CyO-GFP*	VDRC 20166
LimK1	*w* limk1*^{2}	BDSC 59033
MAPk-Ak2	*y1 sc* v1 sev21; P{y+t7.7 v+t1.8=TRiP.HMS04456}attP40*	BDSC 57013
Mei-41	*mei-41*^{29D}*/FM7-GFP*	[98]
Mnb	*P{KK102642}VIE-260B*	VDRC 107066
Msn	*P{KK108948}VIE-260B*	VDRC 101517
Msn	*P{VSH330049}attP40*	VDRC 330049
Msn	*w*; msn102 P{ry+t7.]=neoFRT}80B/ TM6B*	BDSC 5945
Niki	*y1 sc* v1 sev21; P{y+t7.7 v+t1.8=TRiP.HMS01477}attP2*	BDSC 35735
Par-1	*y1 w67c23; P{w+mC=lacW}par-1k06323/CyO-GFP*	BDSC 10615
Pdk1	*P{KK108363}VIE-260B*	VDRC 109812
Pdk1	*y1 v1; P{y+t7.7 v+t1.8=TRiP.JF02807}attP2*	BDSC 27725
Pkc53E	*w1118; Pkc53EDelta28*	BDSC 80988
Pkc53E	*y1 v1; P{y+t7.7 v+t1.8=TRiP.JF02641}attP2*	BDSC 27491
Pkc53E	*y1 sc* v1 sev21; P{y+t7.7 v+t1.8=TRiP.HMS01195}attP2*	BDSC 34716
Pkc98E	*w1118; PBac{w+mC=WH}Pkc98Ef0622]/TM6B, Tb1*	BDSC 18950
Pkc98E	*y1 v1; P{y+t7.7 v+t1.8=TRiP.JF02470}attP2*	BDSC 29311
Pkc98E	*y1 sc* v1 sev21; P{y+t7.7 v+t1.8=TRiP.GL00174}attP2*	BDSC 35275
PKD	*PKD*^{cl4}	BDSC 93864
PKD	*y1 v1; P{y+t7.7 v+t1.8=TRiP.JF03144}attP2*	BDSC 28717
Pll	*P{KK102624}VIE-260B*	VDRC 103774
Put	*y1 v1; P{y+t7.7 v+t1.8=TRiP.JF02664}attP2*	BDSC 27514
Raf	*y1 wa Raf12/FM7-GFP, wi Myc+*	BDSC 5779
S6k	*y1 w*; S6kl-1/TM6B, P{y+t7.7ry+t7.2=Car20y}TPN1, Tb1*	BDSC 32552

Sgg	*sgg^{M1-1}/FM7-GFP*	BDSC 5402
Sik2	*y1 v1; P{y+t7.7 v+t1.8=TRiP.HMC04153}attP2*	BDSC 55880
Slpr	*y1 w* sn3 slpr3P5 P{w+mW.hs=FRT(whs)}101/ FM7a*	BDSC 58795
Slpr	*w1118 slprBS06 P{ry+t7.2=neoFRT}19A/ FM7c, sn+*	BDSC 58807
Slpr	*P{KK100726}VIE-260B*	VDRC 106449
Slpr	*w1118; P{GD9771}v33518/CyO*	VDRC 33518
Tefu	*y1 sc* v1 sev21; P{y+t7.7 v+t1.8=TRiP.HMS02790}attP40*	BDSC 44073
Tkv	*tkv^1*	BDSC 427
Wee1	*y1 v1; P{y+t7.7 v+t1.8=TRiP.HMC03331}attP40*	BDSC 51776
Wnk	*y1 v1; P{y+t7.7 v+t1.8=TRiP.HMJ02087}attP40/ CyO-GFP*	BDSC 42521

Supplemental Table S2

List of oligonucleotides used for constructs

pBT-HA-STaRT

**HA-STaRT Acc_UP**	5' GTA CC**A TG***T ATC CCT ATG ATG TGC CAG ACT ATG CT*G GC*T ATC CAT ATG ATG TTC CTG ATT ATG CT*G GAT *ACC CTT ATG ATG TGC CAG ACT ATG CCC* 3'
**HA-STaRT Xho_LP**	5' TCG AG*G GCA TAG TCT GGC ACA TCA TAA GGG TA*T CC*A GCA TAA TCA GGA ACA TCA TAT GGA TA*G CC*A GCA TAG TCT GGC ACA TCA TAG GGA TA***C AT**G 3'

Cdk7

***Xho*I_Cdk7_UP**	5' TAT CAC TCG AGA TGC TGC CCA ATG 3'
**Cdk7_*Xba*I_LP**	5' GAA TGG TCT AGT CTA GAT TAG AAC TGC AGC C 3'

Dyrk3

***Sal*I_Dyrk3_UP**	5' TAA GGT CGA CAT GGT TGG TTC TCA AGA 3'
**Dyrk3_*Bam*HI_LP**	5' CAT TGG ATC CCT ACA TGT CCT TCG ATT 3'

Fray

***Sal*I_frayed_UP**	5' TAT CAG TCG ACA TGA CCT CCA TAC CC 3'
**frayed_*Eco*RI_LP**	5' TAT TGA ATT CCT AGT CCG TGA TGG AGA T 3'

Gskt

***Xho*I_gskt_UP**	5' ATC ACT CGA GAT GGC TTC CCA GAG TA 3'
**gskt_*Xba*I_LP**	5' GTC GTC TCT AGA TTA GTT TTC ATC CTC TTC CTC 3'
